# The Effect of Short Sleep Duration on the Development of Asthma

**DOI:** 10.1155/2022/3378821

**Published:** 2022-05-16

**Authors:** Zhigang Hu, Xinyu Song, Ke Hu

**Affiliations:** ^1^Department of Respiratory and Critical Care Medicine, The First College of Clinical Medicine Science, China Three Gorges University, Yichang 443003, China; ^2^Department of Respiratory and Critical Care Medicine, Yichang Central People's Hospital at Zhijiang, Zhijiang 443003, China; ^3^Department of Respiratory and Critical Care Medicine, Yichang Central People's Hospital, Yichang 443003, China; ^4^Department of Respiratory and Critical Care Medicine, Renmin Hospital of Wuhan University, Wuhan, China

## Abstract

Asthma is regarded as a heterogeneous disease with chronic airway inflammation and reversible airway limitation. Asthma itself and recurrent attacks of asthma can decrease sleep duration and increase the prevalence of short sleep duration. Systemic low-grade inflammation and obesity caused by short sleep duration have been known for a long time, which potentially affect the development of asthma. It would be interesting to study the interaction between short sleep duration and asthma. However, there are relatively few studies and no review about the association between short sleep duration and asthma. This article performed a review about the relationships between short sleep duration and asthmatic phenotype, laboratory tests, comorbidity, and clinical outcomes. Pooled studies about short sleep duration and asthma provided following four results: (1) compared with healthy sleep duration, short sleep duration seemingly increased the risk of central obesity in asthmatics; (2) short sleep duration potentially reduced the level of FeNO and increased lung function impairment in patients with asthma; (3) asthmatic comorbidities, mainly obesity and depression, were negatively associated with short sleep duration among asthmatics; (4) short sleep duration potentially increased the risks of asthma-related hospitalization and emergency care. However, almost all studies are based on subjective but not objective sleep duration. In addition, the study on sleep duration and cause-specific mortality in patients with asthma is relatively scant. Considering the effect of short sleep duration on the development of asthma, we recommend that periodic sleep monitoring for asthmatic management is very necessary.

## 1. Introduction

Asthma is a common respiratory disease characterized by chronic airway inflammation with reversible airway limitation. Its clinical symptoms are mainly wheezing, chest tightness, dyspnea, coughing, etc. The incidence of asthma ranges from 1% to 18% in the world [1]. Over the past decade, the age-adjusted incidence and prevalence of asthma have, respectively, increased by 2.8% and 3.9% [2]. The emerging evidence confirmed that asthma is not a single chronic inflammatory disease but a heterogeneous disease with multiple phenotypes caused by multiple pathophysiological mechanisms. Recurrent asthmatic attacks can affect the patient's mobility, mental and work status, and quality of life, and severe asthma can even lead to death. The Centers for Disease Control of the United States counted the data about acute exacerbation of asthma from 2011 to 2016. 44.7% of asthmatic patients had an acute exacerbation in the past year, and 9.9% experienced asthma-related emergency care visits. With the increase in public health awareness, long-term and standardized follow-up and drug treatment have well controlled the symptoms of asthma patients and the mortality caused by asthma has decreased by 17.4% in the last decade. Reasonable treatment strategies can control asthma and reduce the burden of asthma.

Sleep is an active process that can restore spirit and reduce fatigue. Adequate sleep, proper exercise, and balanced nutrition are considered to be the three health standards recognized by the international community. Sleep accounts for about a third of a person's life. Previous studies showed that healthy sleep duration plays a key role in an individual's physical and intellectual development, work efficiency, cognitive and mental status, and death. For adults, a healthy sleep duration is recommended at 7-8 h. Numerous epidemiological studies and meta-analyses demonstrated that short sleep duration (<7 h per night) may increase the risks of hypertension, diabetes, metabolic diseases, cancer, proteinuria, cardiovascular diseases, and all-cause mortality [3–7]. In recent years, more attention has been paid to the effect of sleep on allergic diseases. Night sleep plays an important role in congenital and acquired immunity. Obstructive sleep apnea is a disease that naturally affects sleep and is associated with asthma outcomes [8]. Short sleep duration has strong effects on immunity, inflammatory state, and cytokine production. Proinflammatory cytokines in monocytes representing the acute phase response could show an increase in serum levels of IL-6 and TNF-*α* and related mRNA levels after slight sleep deprivation of one night (sleep duration ≤4 h), which remained high after a night-off [9]. Continuously short sleep duration can slightly affect the changes of *T* cytokines, including IL-2, IL-4, and IFN-*γ*. Obesity and low-grade inflammation may be regarded as the crucial mechanisms linking short sleep duration and asthma [10].

**Figure 1:**
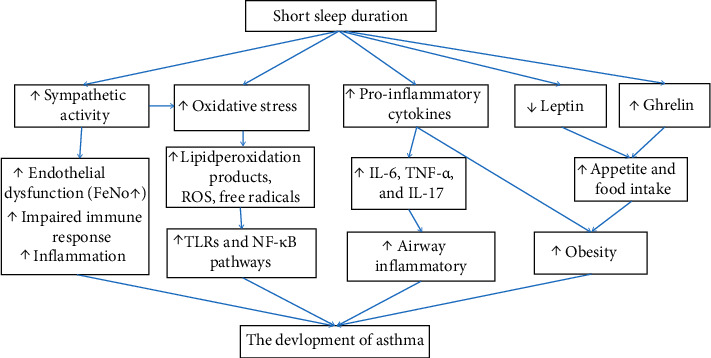
Pathophysiological pathways linking short sleep duration and the development of asthma.

Asthma in stable condition led to a reduction of approximately 50 minutes of sleep duration [11, 12]. Frequent asthmatic attacks also potentially affect sleep quality. GINA2020 clearly points out that it is necessary to evaluate whether asthma causes nocturnal arousal when assessing asthma control levels [1]. In addition, some risk factors of asthma (such as smoking, rhinitis, anxiety, and depression) may lead to difficulty in falling asleep and short sleep duration. The prevalence of short sleep duration (≤6 h) ranged from 45% to 50.8% in three studies about asthmatics [13–15], which seemed to be higher than that in the general population. Our previous study also observed that the prevalence of short sleep duration is gradually increasing with the deterioration of asthmatic clinical outcomes [14]. Short sleep duration may result in some complications such as chronic low-grade inflammation, obesity, and immune dysfunction (see Figure 1), which potentially affect the development and acute exacerbation of asthma and lung function impairment. Asthma and short sleep duration interact with each other. It can be expected that well-controlled asthma will reduce the risk of short sleep duration, and healthy sleep duration also has a positive effect on asthma control and provides adherence to asthma medications. This article will provide a review on the associations between short sleep duration and asthmatic phenotype, laboratory tests, comorbidity, and clinical outcomes.

## 2. Body

### 2.1. The Effect of Short Sleep Duration on Asthmatic Phenotype

In GINA 2020, asthma is broadly classified into five major phenotypes: allergic asthma, nonallergic asthma, adult-onset asthma, asthma with persistent airway limitation, and asthma with obesity [1]. Asthma and chronic obstructive pulmonary disease overlap is a unique type, which is defined as having clinical characteristics of both asthma and chronic obstructive pulmonary disease. Compared with asthma and chronic obstructive pulmonary disease alone, asthma-chronic obstructive pulmonary disease overlap usually required higher medical consumption, harbored more severe respiratory symptoms, and was associated with worse quality of life and more asthma-related hospital visits [16]. In addition, researchers classified asthma into T2-high and T2-low inflammation based on pathophysiological properties [17]. About half of the asthmatics are classified as having T2-high inflammation, and the proportion of T2-high inflammation in severe and refractory asthma patients is higher. T2-high asthma can be roughly divided into allergic asthma and nonallergic eosinophilic asthma according to different mechanisms. It mainly depends on IgE, which plays an important role in allergic asthma. In nonallergic eosinophilic asthma, inflammatory factors (mainly IL-4, IL-5, and IL-13) play a leading role. Asthma with high levels of fractional exhaled nitric oxide (FeNO) and a high blood eosinophil count can be classified as T2-high asthma. Neutrophil inflammation stimulated T1 or T17 cell release in T2-low asthma and induced Muc5ac and Muc5b expression in bronchial epithelial cells, which led to goblet cell proliferation, airway remodeling, glucocorticoid resistance, and the occurrence and development of severe or refractory asthma [18].

Hu et al. evaluated the effects of short sleep duration on the asthma phenotype in 538 patients with current asthma [15]. The results showed that short sleep duration seems to significantly increase the risk of asthma with central obesity but has no significant effects on asthma with general obesity, asthma with persistent airway limitation, and asthma-chronic obstructive pulmonary disease overlap. At the same time, the study also observed that short sleep duration has no significant effect on the prevalence of asthma with high levels of blood eosinophil count (>300 *μ*l) and FeNO (>25 ppb). Nunes et al. assessed the effects of sleep deprivation and healthy sleep on airway and systemic inflammation by using an allergic mouse model [19]. Compared with healthy sleep, sleep deprivation was associated with more severe airway inflammation and higher levels of systemic inflammation (mainly including IL-6, TNF-*α*, and IL-17). The migration of Th17-committed CD4+ T cells to the lung and the increase of neutrophil-related inflammatory mediators secondary to sleep deprivation induced allergic mice to develop neutrophilic lung inflammation through IL-17 signaling and led to corticosteroid-resistance in allergic mice [19].

### 2.2. The Effects of Short Sleep Duration on the Laboratory Test of Asthma

The levels of FeNO, blood eosinophil count, and IgE have some guiding values in the diagnosis and treatment of asthma [1]. Asthmatics with high levels of FeNO, blood eosinophil count, and IgE tend to receive the treatment of inhaled corticosteroid and biologic agents with good therapeutic effects. High level of blood eosinophils was associated with a high risk of asthma-related attacks and emergency room visits [19]. Simultaneously elevated FeNO and blood eosinophil count increased the risk of wheezing symptoms and lung function impairment in asthmatics [20, 21]. The level of lung function can be used to evaluate the severity and phenotype of asthma. A significant decrease in lung function would affect the quality of life of patients and increase the risk of asthma-related exacerbation [1]. Asthma with persistent airway limitation is associated with more clinical symptoms and is more difficult to treat than allergic asthma.

Meltzer et al. completed a three-week randomized controlled study, which firstly assessed the effects of sleep duration on the levels of lung function and FeNO in adolescents with asthma [22]. Compared with healthy sleep duration, short sleep duration reduced the levels of FEV1 and PEF by 14% and 6%, respectively, but had no significant effect on the level of FeNO. However, the sample size of the study with only 10 patients was relatively small. Another study based on 216 children showed no significant correlation between sleep duration and the change of FEV1 [23]. We simultaneously evaluated the effects of short sleep duration on the levels of lung function, FeNO, blood eosinophil count, and percentage in adults with current asthma [15]. Compared with healthy sleep duration (7-8 h), short sleep duration (≤6 h) was associated with the significant increase of blood eosinophil percentage (adjusted OR = 1.19, 95% CI: 1.14–1.26, *P* < 0.01) and the reduction of FeNO level (adjusted OR = 0.86, 95% CI: 0.77–0.96, *P* < 0.01) [15]. In addition, we also observed that asthmatics with short sleep duration harbor worse lung function than those with healthy sleep duration. The most significant difference in lung function between the short and healthy sleep duration groups was FEF 25%–75% (adjusted OR = 1.051, 95% CI: 1.047–1.055), followed by FEV1 (adjusted OR = 1.020, 95% CI: 1.016–1.023) and FVC (adjusted OR = 1.016, 95% CI: 1.013–1.019). Short sleep duration seemingly had no significant effect on FEV1/FVC (adjusted OR = 1.008, 95% CI: 0.821–1.239, *P*=0.93).

### 2.3. The Effect of Short Sleep Duration on Comorbidity of Asthma

The presence of comorbidity in asthmatics potentially increases the risk of recurrent asthma symptoms, reduces quality of life, and sometimes leads to poor asthma control. Previous studies noted the effects of comorbidities in patients with asthma [1, 24], which mainly included obesity, depression and anxiety, rhinitis, rhinosinusitis, gastroesophageal reflux, and obstructive sleep apnea (see Table 1).

Asthma with obesity has been identified as a unique phenotype of asthma, which is more prone to T2-low inflammation (mainly neutrophilic asthma). Asthma with obesity was more difficult to manage and was often accompanied by worse lung function compared with allergic asthma. A large number of cross-sectional and longitudinal studies suggested that short sleep duration significantly increases the risk of obesity. A meta-analysis showed that short sleep duration increases the risk of developing obesity by 41.2% compared to healthy sleep duration (7-8 h) [25]. Patel et al. summarized several potential mechanisms to explain the association between sleep duration and obesity [26]: (1) short sleep duration means there is an increase in waking time and provides more opportunities for eating; (2) short sleep duration can stimulate secretion of polypeptide ghrelin by increasing stomach-derived appetite, which reduces satiety and increases hunger; (3) short sleep duration may increase hypothalamic-pituitary-adrenal activity, affect glucose metabolism homeostasis, and cause metabolic dysfunction; (4) short sleep duration can increase fatigue, which also means that people with short sleep duration are often accompanied by lower levels of physical activity. The reduction of physical activity potentially increases the risk of obesity. Compared with general obesity, central obesity was known to be more available to reflect body fat distribution and measure the associations between obesity and other diseases [10]. Our previous study observed that short sleep duration (≤6 h) in adult asthmatics is associated with the significant increase in developing asthma with central obesity than healthy sleep duration (7-8 h) but has no correlation with the risk of asthma with general obesity [15]. In another study, self-reported sleep duration was divided into five groups: <6 h (very short), 6 to <7 h (short), 7-8 h (normal), >8 to ≤9 h (long), and >9 h (very long) [27]. With reference to normal sleep duration, short sleep duration (adjusted OR = 1.66, 95% CI: 1.07–2.57) was associated with the significantly increased risk of asthma with general obesity in the final model. The association between very short sleep duration and asthma with general obesity approached significance (adjusted OR = 1.74, 95% CI: 0.96–3.14, *P*=0.06). The increased risk of obesity was associated with the improvement of asthma (adjusted OR = 1.87, 95% CI: 1.09–3.21) and a tendency for high-dose inhaled corticosteroids (adjusted OR = 1.82, 95% CI: 0.93–3.56).

The effects of anxiety and depression on symptom control scores, lung function, and body mass index were evaluated in patients with asthma [28]. The results showed that the symptom control scores of asthmatics with anxiety are significantly lower than those of asthmatics without anxiety disorder (18.3 vs. 20.9, Spearman *r* = −0.37, *P*=0.001). Asthmatics with depression also had significantly lower asthma symptom control scores than those without depression (16.5 vs. 20.4, Spearman *r* = −0.35, *P*=0.001). Patel et al. assessed the association between depression and asthma-related attacks on the basis of the National Health and Nutrition Survey Database (2007–2012) [29]. The results showed that depression potentially increases 1.53 times (95% CI: 1.00–2.35) in asthma-related attacks, 2.24 times (95% CI: 1.15–4.34) in asthma-related emergency room visits, 2.75 times (95% CI: 1.54–4.92) in sleep disorders, and 1.77 times (95% CI: 1.00–3.18) in activity restriction, respectively. Patients with asthma and depression were associated with worse general health, physical and mental health, and physical activity than those without depression [29]. Short sleep duration is likely to increase the risk of developing anxiety and depression through the following mechanisms: (1) short sleep duration results in daytime physical and psychological fatigue, which may destroy circadian rhythms and cause changes in hormone levels in the body, thus leading to the incidence of depression; (2) chronic low-grade inflammation secondary to short sleep duration may be a key biological pathway to induce depressive symptoms. The study in Chinese population demonstrated that middle-aged people (45–65 years) with short sleep duration (<6 h) are more likely to be associated with depressive symptoms (adjusted OR = 1.45, 95% CI: 1.193–1.764) than those with healthy sleep duration [30]. Meanwhile, the risk of depressive symptoms in the short sleep duration group increased 2.08 times (95% CI: 1.479–2.936) than that in the healthy sleep duration group for the elderly population (≥65 years) [30]. Another study evaluated the effects of sleep duration on the prevalence and severity of depression in the nursing population [31]. Compared to sleep duration of ≥8 h, 6-7 h of sleep duration had an OR value of 1.6 (95% CI: 1.2–2.2) for any depressive symptoms, 1.5 (95% CI: 1.1–2.2) for mild to moderate depressive symptoms, and 1.9 (95% CI: 1.1–3.4) for severe to extremely severe depressive symptoms. For nurses who sleep ≤5 h, the OR with any depressive symptom was 2.1 (95% CI: 1.4–3.3), the OR with mild to moderate depressive symptoms was 1.7 (95% CI: 1.0–2.6), and the OR with severe to extremely severe depressive symptoms was 4.2 (95% CI: 2.2–8.1) with reference to 8 h sleep. Overall, short sleep duration not only affects the risk of depressive symptoms but also influences the severity of depressive symptoms. In patients with asthma, only one study estimated the association between short sleep duration and depressive symptoms [32]. Compared with normal sleep duration, short sleep duration seemed to significantly increase the risk of depressive symptoms (adjusted OR = 1.26, 95% CI: 1.11–1.44). Our previous study about the association between sleep duration and asthmatic episodes/attacks had the data about depression in asthmatics [14]. Secondary analysis was performed to determine the difference in depression between short and healthy sleep duration in asthmatics. Compared with healthy sleep duration (7-8 h), the prevalence of depression in asthma patients with short sleep duration (≤6 h) was significantly higher (adjusted OR = 1.41, 95% CI: 1.10–1.80). With reference to 7 h, the reduction in sleep duration was associated with a gradual increase in depression.

One study involving 274,480 adolescents aged 12 to 18 years assessed the association between sleep duration and allergic rhinitis [33]. Sleep duration of <7 h and sleep after 24:00 were also likely to increase the risk of developing allergic rhinitis. Another study also observed that short sleep duration increases the risk of allergic rhinitis by 1.53 times (95% CI: 1.11–2.11) after adjusting for confounding factors [34]. The study in South Korean adults showed that sleep duration may have a negative relationship with chronic rhinosinusitis [35]. There is no published study about the relationships between short sleep duration and rhinitis and rhinosinusitis in patients with asthma. Gastroesophageal reflux potentially shortened sleep duration. However, whether short sleep duration increases the risk of gastroesophageal reflux remains for further study. In addition, we found no study to assess the effect of short sleep duration on the development of obstructive sleep apnea in asthmatics.

### 2.4. The Effect of Short Sleep Duration on Asthma-Related Symptoms and Acute Attacks

Risk factors affecting acute exacerbation of asthma are identified in GINA2020 [1], including any patient with ≥1 risk factor for exacerbations (including poor symptom control and infection), a previous history of ≥1 severe exacerbation in the last year, persistent tobacco exposure, low lung function (especially if FEV1 <60% predicted), obesity, major psychological problems (anxiety and depression), major socioeconomic problems, confirmed dietary allergy, allergen exposure, and eosinophilia in the sputum (see Table 1).

The associations between short sleep duration and obesity, depression, and lung function impairment in patients with asthma have been formulated in the front part. Short sleep duration also potentially affects the following risk factors: infection and allergen exposure. Short sleep duration may reduce the body's immune system by multiple mechanisms, which include lymphopenia, the reduction of proliferation of T cells, downregulation of leukocyte antigen-DR homotypic expression, and disproportionate alterations in natural killer, helper, and cytotoxic T cells [36]. It can be expected that short sleep duration will make individuals more susceptible to viruses and bacteria, thus increasing the risk of viral infection and pneumonia. Two studies demonstrated that participants with a sleep duration of less than 7 hours have a significant increase in the risk of developing a clinical cold compared with sleep duration ≥8 h per day [37, 38]. In another study, women who slept ≤6 h per night were associated with an increased risk of pneumonia (adjusted OR = 1.38, 95% CI: 1.06–1.82) than those who slept 8 h per night [39]. Short sleep duration also potentially affects the sensitization of allergens. In the study involving 1,534 rural Chinese teenagers (aged 12–21 years), sleep duration was divided into three groups according to population distribution: 3.0–7.8 h (short), 7.8–8.9 h (normal), and 8.9–14.7 h (long). Compared to the long sleep duration group, the risk of food allergy significantly increased in both the short (OR = 1.9, 95% CI: 1.3–2.7) and normal (OR = 1.4, 95% CI: 1.0–1.9) sleep duration groups [40]. The number of positive skin prick tests demonstrated a significant dose-response relationship with the percentage of short sleep duration.

The study by Teodorescu et al. found that very short habitual sleep (<6 h) is associated with higher asthma steps (OR = 2.77, 95% CI: 1.71–4.49) and more persistent (step ≥ 2) asthma (OR = 2.69, 95% CI: 1.53–4.71) [27]. Two existing studies assessed whether short sleep duration affects the risk of asthma-related symptoms and acute attacks [14, 41]. Compared to normal sleep duration (6–8 h), short sleep duration (≤5 h) potentially increased the risks of asthma-related attacks (adjusted OR = 1.58, 95% CI: 1.13–2.21), asthma-related cough (adjusted OR = 1.95, 95% CI: 1.32–2.87), and asthma-related hospitalization (adjusted OR = 2.14, 95% CI: 1.37–3.36) [41]. In addition, short sleep duration had negative effects on the patient's daily life, mental health, and activity ability. Another study involving 1,526 asthmatics assessed the effects of short sleep duration on the prevalence of asthma-related attacks and emergency visits and the number of asthma-related emergency care visits [14]. Compared with short sleep duration (≤6 h), healthy sleep duration (7-8 h) was associated with a significant reduction in the prevalence (adjusted *RR* = 0.67, 95% CI: 0.47–0.94) and number (adjusted *RR* = 0.83, 95% CI: 0.69–0.9996, *P*=0.0495) of asthma-related emergency care visits.

### 2.5. The Problem and Prospect of Short Sleep Duration and Asthma

It should be noted that sleep duration includes subjective and objective sleep duration. Subjective sleep duration is mainly obtained by self-report, while objective sleep duration is detected by polysomnography and actigraphy. The study showed moderate consistency between subjective and objective sleep duration (*r* = 0.28, *P*=0.0001) [42]. Objective sleep duration was 30.7 minutes less than subjective sleep duration, and this difference was more pronounced in patients with insomnia [40]. Studies showed that subjective and objective sleep duration can have different clinical effects in the same population [43, 44]. The impact of sleep duration on disease is optimally measured by objective sleep duration, whereas the results based on subjective sleep duration can be regarded as a validation and supplement to the study.

To date, the majority of current studies about sleep duration and asthma were based on subjective sleep duration. The effects of objective short sleep duration on asthmatic phenotype, laboratory tests, comorbidity, and clinical outcomes remain to be further explored. In addition, the study about the associations between short sleep duration and asthma-related death and all-cause mortality is relatively few. Based on the Sleep Heart Health Study, we performed the analysis with a multivariate Cox regression model and found that objective but not subjective sleep duration is associated with the increased risk of all-cause mortality among asthmatics. Hospitalization or emergency care visits for asthma in the past year and a history of psychiatric disease or psychosocial problems are also risk factors of increasing asthma-related deaths [1]. It is expected that short sleep duration has the ability to cause asthma-related death by increasing the risk of asthmatic hospitalization or emergency care visits and depression. More large-sample prospective studies are warranted to determine the association between short sleep duration and cause-specific mortality in patients with asthma and deeply study the potential interaction between asthma and subjective and objective short sleep duration.

Melatonin was found to harbor antioxidant and cytoprotective effects in several inflammatory conditions, which resulted in important immunostimulatory actions in allergic diseases [45]. In addition, animal experiments suggested that melatonin treatment may ameliorate inflammatory cell infiltration, the levels of serum IgE, Th2, and Th17 cytokines in BALF, obvious changes in lung histology, and hyperresponsiveness by inhibiting the TRPV1 channel and stabilizing the Nrf2 pathway [46]. A randomized controlled study involving 22 females with mild or moderate asthma demonstrated that melatonin treatment as a sleep promoter agent has no significant effects on pulmonary function, asthma symptoms, and use of relief medications but significantly improves subjective sleep quality [47]. However, this study had the following limitations: firstly, the study population with relatively few samples only focused on mild or moderate asthma; secondly, all the included asthmatics were female. Further large-sample studies about the long-term effects and dose of melatonin on asthma management are necessary.

## 3. Conclusions

Short sleep duration potentially affects the development of asthma with obesity and neutrophilic asthma. Lung function impairment and the reduction of FeNO level may be caused by short sleep duration in asthmatics, which in turn increases the difficulty of treating and controlling asthma. Short sleep duration may increase the difficulty of asthmatic management by affecting comorbidity and risk factors of acute attacks such as inflammation, obesity, depression, infection, and allergen exposure. Therefore, we believe that periodic monitoring of sleep duration is very necessary for the management of asthma, and even asthma with a short sleep duration may be considered as a new phenotype of asthma.

## Figures and Tables

**Table 1:** Risk factors related to asthma control, exacerbations, and death.

(A) Risk factors related to asthma control
Irrational drug use
Exposures (smoking, allergen exposure, and air pollution)
Low lung function (especially FEV 1 < 60%)
Rhinitis
Rhinosinusitis
Gastroesophageal reflux
Obesity
Obstructive sleep apnea
Major psychological or socioeconomic problems (depression and anxiety)
Confirmed food allergy
Type 2 inflammation
History of asthma exacerbations

(B) Risk factors related to asthma exacerbations
Viral respiratory infections
Allergen exposure
Food allergy
Outdoor air pollution
Seasonal changes
Poor adherence to ICS
Epidemics of severe asthma exacerbations

(C) Risk factors related to asthma deaths
A history of near-fatal asthma requiring intubation and mechanical ventilation
Hospitalization or emergency care visit for asthma in the past year
Currently using or having recently stopped using oral corticosteroids
Not currently using inhaled corticosteroids
Overuse of SABAs
Major psychiatric disease or psychosocial problems (depression and anxiety)
Poor adherence to asthma medications
Food allergy in a patient with asthma

The contents of the table are in references to GINA 2020.

## Data Availability

No data were used to support this study.
